# College of Physicians of Philadelphia

**Published:** 1886-01

**Authors:** 


COLLEGE OF PHYSICIANS, OF PHILADELPHIA.

*Stated meeting, October 7.*

*Cocaine in Hay-Fever.*—A report on the influence of cocaine in cases of rose-cold and hay-fever was read by Dr. J. M. Da Costa, supplementing the writer's claims in his former paper of last year. The success which has attended this method of treatment, in his own experience and that of others, induces him to recommend it highly for all ordinary cases.

Solutions of less than four per cent. are insufficient, and some cases require eight. per cent. of the salt to produce noticeable improvement. The liquid may be thrown into the nostrils in the form of spray, but better results are obtained when five to eight drops are instilled with a medicine-dropper.

The distressing sneezing and the subsequent asthmatic attacks of hay-fever are prevented, entirely, by this means in some cases. Bronchial catarrh is not much benefited.

A certain insusceptibility—in the author's experience—or a varying susceptibility, is seen in the local use of cocaine. It is to be observed that some patients fail to receive benefit from its application.

A single case is mentioned in which increased vascular tension and severe headache followed its use in four per cent. solution.

Its mode of action in hay-fever is partly by the local insensibility it produces, partly by the contraction of the capillaries it induces.

That it is a radical, and, strictly-speaking, curative remedy, cannot be maintained, but it has been fairly tested and proven to give great comfort, and to convert very bad cases into light ones. In no case of rose-cold or hay-fever ought cocaine to be left untried. It will be found that many who employ it will be able to remain at their homes, who otherwise would be obliged to flee to hay-fever resorts.

In the discussion of this paper Dr. Harrison Allen confirmed the statement of the writer, that the effect of cocaine is inconstant, within a narrow range, in different individuals. In endeavoring to account for this, he advanced the theory that a difference in the erectile tissue in the nostrils may be the real cause. Those persons in whom the tissue is sparsely developed may be less susceptible to impression by the remedy than those in whom it is well developed.

Examples were cited in which the nasal membranes were quite insensitive to any treatment, such as galvanism, which ordinarily constricts the capillary network. These are the cases which fail to respond to cocaine. A majority of cases are relieved by it, however.

Dr. H. C. Wood called attention to the observations of Dr. Lyons, of Detroit, wherein it was shown that there are two or three alkaloids in coca-leaf, and that commercial cocaine not rarely is composed of more than one alkaloid. Ecgonine and perhaps one other alkaloid are present at times.

The unexpected therapeutic results from the use of cocaine may possibly be due to the presence of one of these alkaloids.

In response to the question whether caffeine is an agent capable of being substituted for cocaine, as an anæsthetic, Dr. Wood answered that he had made experiments with caffeine on the eyes of patients, and found it without effect.

*Treatment of Sunstroke in the Pennsytvania Hospital.*—A record of fifty cases of sunstroke treated at Pennsylvania Hospital was then read by Dr. Orville Horwitz, resident-physician of the institution.

The minimum temperature in any case was 112°, and this patient recovered.

*Antipyrin*, in large doses, was used in all cases of pyrexia, in conjunction with ice-bags and ice-sheets to the surface.

*Musk* was found efficacious in controlling convulsions.

*Aqua Ammonia* doubtless saved several cases where the patient was about to die of heart-failure.

*Ether*, hypodermically, acted as a better stimulant than whisky.

*Blood-letting*.—One individual was bled from the arm. He died two days after, from meningitis.

Four persons were cupped at the nape of the neck. They all recovered.

All these persons were full-blooded—heavy men, with injective conjunctiva; the veins were promineut in the neck, and there was full, bounding pulse, with convulsions setting in early.

*Dry cups*, true sunstroke, were valueless but in heat—exhaustion, the benefit was well marked.

*Digitalis* acted as an excellent heart-stimulant.

*Quinine*, following the antipyrin, was of great benefit.

*Calomel*, qr. x, and bromide of sodium, qr. xxx, were given when consciousness had been restored.

Of these cases, *twenty-four* were cases of sunstroke, and *twenty-six* suffered from heat exhaustion.

Of the *twenty-four* cases of sunstroke, *nine died. Three died* within *ten minutes* after admission, and cannot fairly be said to have been subjected to treatment in the institution.

*Four died* within *six hours* after admission. *Two died forty-eight hours* after admission.

Of the nine that died, *four were hard drinkers; two were strictly temperate*, and *three drank in moderation.* Twenty-one out of the twenty-four had violent convulsions; one had acute mania, lasting one hour and a half.

The maximum temperature was 112° F.; this patient recovered.

The minimum temperature was 94 2-5°; this was a case of heat exhaustion.

Twenty out of the twenty-four cases of sunstroke occurred between July 16 and July 26.

The largest number received on any one day was on Wednesday, July 22, when nine cases were admitted. The thermometer on that day stood at $93\frac{5}{10}$° F. in the shade; on the two previous days it rose to 100° F. in the shade.

But two opportunities for post-mortem examinations presented themselves. In one case, which resulted in death ten minutes after admission, the temperature being 109° F., congestion of the lungs and kidneys was found to exist, with slight injection of the arachnoid and pia mater. In the remaining case, the individual dying two days after admission, there were presented the usual evidences of commencing meningitis.

On examination of the blood, the corpuscles were found shriveled in a few cases, but in the majority the microscope revealed no change.

Albumen was present in the urine in all but two cases, and this condition continued for two or three days after convalescence.

Dr. C. H. Wood said: The use of musk, as detailed in the paper just read, is, I believe new. Antipyrin has, however, been used in one of the New York hospitals, and a paper written thereon by the resident physician.

There is one point which is worthy of consideration by hospital authorities. I have noticed myself, in experiments on animals, that time is of the utmost importance in the treatment of sunstroke, and our clinical experience accords with this. If the moment the animal became unconscious, I reduced the temperature by cold, the animal invariably recovered; if, however, it was left for ten or twenty minutes, reduction of the temperature caused benefit, and usually return of conciousness, but there were almost always marked signs of an impaired nervous system, and in a large proportion of cases death from paralysis. In the New York Hospital, antipyrin was given to the ambulance surgeon, and thus the remedy could be administered at once. I myself think that in very hot weather the hospital ambulance should be provided, not only with antipyrin, but also with ice, and no time would be lost, the remedies being applied as the patient was being brought to the hospital. The patient could be half undressed and rubbed with ice, and antipyrin could be used hypodermically.

